# Conservation tillage practices affect soil microbial diversity and composition in experimental fields

**DOI:** 10.3389/fmicb.2023.1227297

**Published:** 2023-08-02

**Authors:** Muzammil Hassan Khan, Hao Liu, Anning Zhu, Mudassir Hassan Khan, Sarfraz Hussain, Hui Cao

**Affiliations:** ^1^College of Life Sciences/Key Laboratory of Agricultural Environmental Microbiology, Ministry of Agriculture, Nanjing Agricultural University, Nanjing, China; ^2^Fengqiu Agro-Ecological Experimental Station, State Key Laboratory of Soil and Sustainable Agriculture, Institute of Soil Science, Chinese Academy of Sciences, Nanjing, China; ^3^Department of Biological Sciences, Karakoram International University, Gilgit, Pakistan; ^4^Key Laboratory of Integrated Regulation and Resource Development on Shallow Lakes of Ministry of Education, College of Environment, Hohai University, Nanjing, China

**Keywords:** conservation tillage, soil properties, soil bacterial community structure, Illumina sequencing, KEGG pathway, bacterial network analysis

## Abstract

**Introduction:**

Conservation tillage is a widely used technique worldwide, but the effects of conservation tillage on bacterial community structure are poorly understood. We explored proportional alterations in the bacterial community under different tillage treatments.

**Methodology:**

Hence, this study utilized high-throughput sequencing technique to investigate the structure and assembly processes of microbial communities in different tillage treatments.

**Results and discussion:**

Tillage treatments included tillage no-straw retention (CntWt), no-tillage with straw retention (CntWntS), tillage with straw retention (CntWtS), no-tillage and no-straw retention (CntWnt). The influence of tillage practices on soil bacterial communities was investigated using Illumina MiSeq sequencing. Different tillage methods and straw retention systems significantly influenced soil parameters such as total potassium and pH were not affected by tillage practices, while straw retention significantly affected soil parameters including nitrogen content, available phosphorus and available potassium. Straw retention decreased bacterial diversity while increased bacterial richness. The effect of straw retention and tillage on bacterial communities was greater than with no tillage. Phylogenetic β-diversity analysis showed that deterministic homogeneous selection processes were dominated, while stochastic processes were more pronounced in tillage without straw retention. Ecological network analysis showed that microbial community correlation was increased in CntWntS and CntWnt. Straw retention treatment significantly increased the relative abundance of bacterial taxa Proteobacteria, Bacteroidetes, and OD1, while Nitrospirae, Actinobacteria, and Verrucomicrobia significantly decreased.

**Conclusion:**

The conservation tillage practices significantly affect soil properties, bacterial composition, and assembly processes; however, further studies are required to investigate the impact of different crops, tillage practices and physiological characteristics on bacterial community structure and functions.

## 1. Introduction

Agriculture is an important anthropogenic contributor to the variation of soil's physicochemical properties. These changes in soil properties proportionally affect the soil microbial community structure and composition (Kladivko, 2001). The relationship between soil and bacterial communities is an important sign of soil quality, sustainability of agriculture, and the ecosystem (Wang et al., 2016). Fertile soil in farmland is the prerequisite and foundation of sustainable agriculture development (Parikh and James, 2012). Tillage treatments affect soil biogeochemistry and help improve soil fertility (Frey et al., 1999; Gathala et al., 2011). No-tillage is an alternative to conventional tillage, and it usually increases the aggregation of soil organic matter (SOM) and soil biota (Six et al., 2000) and reduces soil erosion and production expenses by saving fuel, equipment, and labor (Uri, 1999; Huang et al., 2013). Soil microbial communities are more abundant in no-tillage soil than in conventional tillage (Govaerts et al., 2007; Helgason et al., 2009). Many Chinese farmers still use traditional tillage methods, especially in the Huang-Huai-Hai Plain in China, including deep plowing and rotational tillage (Chen et al., 2017). These practices are more costly, especially deep plowing (Wang et al., 2006). Nevertheless, tillage increases the bulk density of the soil up to a layer of 10–30 cm compared with no cultivation (Šíp et al., 2013). In addition, rotary tillage reduces the plow layer, enriches surface nutrients, and depletes deeper soil, causing soil infertility and preventing plant uptake of nutrients (Tian et al., 2012; LiangPeng et al., 2015).

Retention of crop straw stimulates microbial activity and biomass, reducing handling costs and time and preventing environmental pollution (Rodriguez et al., 2017). Studies have shown that availability of crop residues increased soil organic carbon (SOC), nitrogen (N) stocks (Brady and Weil, 1996; Dolan et al., 2006; Govaerts et al., 2006; Ke and Scheu, 2008), and microbial activity and decreased soil temperature (Sarrantonio and Gallandt, 2003; Wang et al., 2011). However, inappropriate straw incorporation methods could degrade soil structure and cause an imbalance in the nutrient supply (Kong, 2014; Chen et al., 2017). The fate of crop residues, which provide a diverse substrate, can significantly affect bacterial communities in the soil (Su et al., 2017). The impact of tillage on soil bacterial diversity and assemblages studied so far differs significantly between the different tillage methods (Souza et al., 2016; Dong et al., 2017).

With the recent advancement in deep sequence methods, several studies showed different findings about the impact of tillage and no-tillage practices on microbial community composition and structure. For example, several investigations have found consistent microbiome diversity in soil samples collected under different tillage practices (Hartman et al., 2018; Xia et al., 2019; Kraut-Cohen et al., 2020). Similarly, some studies indicated higher microbial alpha diversity in no-tillage soil than in tillage practices, and some also reported higher biomass richness in no-tillage samples (Feng et al., 2003; Sengupta and Dick, 2015; Wang et al., 2017; Schmidt et al., 2018). According to another research, appropriate post-harvest residue management practices can help restore soil nutrients by decomposing organic residues, thereby reducing the decline of microbial diversity in plantation soils (Chen and Xu, 2005; Xiong et al., 2021). To date, all research endeavors have investigated the impacts of diverse management practices on soil nutrients, long-term site sustainability, plant survival and growth, microbial composition, and several other pertinent factors. Nevertheless, prior research has not investigated the impact of distinct residue management and tillage practices on soil-related microbial community composition and assembly processes.

Numerous studies have shown that straw incorporation significantly affects nitrogen use and crop production (Duan et al., 2014). Both no-tillage and conventional tillage with residues applied had higher total nitrogen uptake and crop production than the no-residue methods applied in the lower middle Yangtze River Delta in China (Xu et al., 2010). In Central China, the yield was significantly higher under deep tillage with crop residues at a depth of 30 cm than without the incorporation of straw (Zhao et al., 2014; Chen et al., 2015). However, in northwest China, no significant difference in nitrogen uptake was found between treatments with and without straw return under no-tillage conditions (Zhang et al., 2009). The effects of tillage on crop production and nitrogen application differ depending on local climate, soil conditions, residue management, and crop rotation (Ponnamperuma, 1984). However, there is still a gap in knowledge about how straw retention affects soil microbial communities under both tillage and no-tillage. In contrast to microbial taxonomic composition, recent investigations have demonstrated that microorganisms' functional composition is more sensitive to environmental variables (Song et al., 2021). Therefore, it would be crucial to characterize the functional profiles of microbial communities to at least partially describe their metabolic properties (Gibbons et al., 2017). Xenobiotic substances are exotic substances that accumulate in the environment and endanger the biosphere. Xenobiotic substances cannot be biodegraded because they are extremely resistant. Although metagenomics technologies are widely used to reveal the activities of microbial communities, their widespread application is limited by their high cost and extremely difficult data processing (Louca et al., 2016). Tillage and residue management practices can impact the availability of nutrients such as carbon and nitrogen contents in soil (Kraut-Cohen et al., 2020). Therefore, in this study we performed PICRUSt, a system that predicts metagenomes using 16S data and a reference genome database using evolutionary modeling. Based on the relatively abundant bacterial sequences in all samples, most predicted metabolic pathways were classified into five functional groups: metabolism, environmental information processing, genetic information processing, cellular processes, and unclassified.

The tillage system produces soil disturbance at different levels, thus creating diverse soil ecosystems that affect bacterial community structure (Navarro-Noya et al., 2013; Sun et al., 2018). Soil crop cultivation conservation includes incorporating no-tillage, crop straw returning, and crop rotation, which is not well-defined and often interpreted diversely (Hobbs et al., 2008; Palm et al., 2014). In this study, we hypothesized that tillage decreases microbial diversity and richness, while straw retention increases microbial diversity and richness. Furthermore, microbial assembly processes were driven by tillage than straw retention in typical fluvo-aquatic soils. This study will help to interpret relationships between soil variables and changes in bacterial community structure and composition under different tillage practices.

## 2. Materials and methods

### 2.1. Treatments and experimental design

We explained the experimental site and sampling strategy in our recently published study (Zhang et al., 2022). In brief, the long-term conservation field was commenced in 2006 and based on a completely randomized block design with three replications. Each treatment plot was 14 m × 6.5 m, and a maize–wheat rotation system was arranged randomly. Four treated soil samples are as follows: (1) tillage with no-straw retention (CntWt), (2) no-tillage with straw retention (CntWntS), (3) tillage with straw retention (CntWtS), and (4) no-tillage with no-straw retention (CntWnt) as control. Regarding tillage practice, soils were plowed to 20–22 cm depth with a moldboard plow. Regarding straw mulching, residues were crushed into 2–3 cm pieces for maize and 6–7 cm pieces for wheat and then were spread evenly on the soil surface as mulch. The amount of straw was the same in each plot. As for no-straw mulching treatments, all residues were removed from the plots. Each experimental plot was 14 m × 6.5 m in size. Three soil samples were randomly collected from each plot from the surface layer of soil (0–20 cm) using a sterile soil driller. The three samples from each plot were immediately mixed to form a composite soil sample. The composite samples were sieved through a 2-mm sieve to homogenize them and remove plant roots and stones before being transferred to the laboratory for storage at 4°C for measuring soil physicochemical properties and at −80°C for DNA extraction (Sparks et al., 1996).

### 2.2. Extraction of soil microbial genomic DNA

According to the Fast-DNA SPIN kit protocol, the microbial genomic DNA was extracted from 0.5 g soil samples (MP Biomedical, Santa Ana, CA). DNA concentrations were quantified using a NanoDrop ND-2000 (NanoDrop2000, Thermo Scientific, and Wilmington, DE, USA) spectrophotometer according to the manufacturer's procedure.

### 2.3. Bacterial 16s rRNA gene PCR amplification

The bacterial 16S rRNA gene was amplified with universal primers 515F (5′-GTAGTCTGTGCCAGCMGCCGCGG-3′) and 907R (5′-CCGTCAATTCMTTTRAGTTT-3′) that target the V4–V5 regions. Master Mix (20 μl) reaction mixture was prepared using a Taq enzyme (Takara, Japan). PCR amplification reaction conditions were as follows: initial denaturation at 95°C for 3 min, then 30 cycles of 95°C for the 30 s, 55°C for 30 s, 72°C for 1 min, with a final extension step of 72°C for 10 min. The amplified PCR product was verified by 1% agarose gel electrophoresis.

### 2.4. Library construction and Illumina sequencing

The high-throughput sequencing of 16S rRNA described by Caporaso et al. (2012) was used to find comprehensive coverage of bacterial community composition and diversity. The PCR products were sent to the Shanghai Personal Biotechnology Co., Ltd company. The amplified 16S rRNA gene was sequenced using the Illumina MiSeq platform. Complete data sets were deposited in the National Center for Biotechnology Information (NCBI) sequence read archive (SRA) with the accession number from SRR25082107 to SRR25082118.

### 2.5. Phylogenetic diversity and assembly process analysis

We studied phylogenetic structure with null model-based analysis to test the assembly mechanisms of the bacterial populations (Kembel, 2010). In brief, phylogenetic turnover was calculated with 999 random sets, and the abundance-weighted mean nearest taxon distance (MNTD) was determined to analyze phylogenetic clustering in a single sample. Subsequently, to measure the standard effect size of the MNTD (ses.MNTD), the command “ses.mntd” in the “picante” package of R was applied (Webb et al., 2002; Stegen et al., 2012). Then, the β-nearest taxon index (βNTI), representing the difference between the βMNTD and the mean of the null distribution of βMNTD normalized by its standard deviation, was calculated to identify the community assembly processes further. The βMNTD and βNTI were calculated in R with the package MicEco using the command ses.comdist and ses.comdistnt, respectively (Zhang et al., 2019). Calculated βNTI values were used to examine phylogenetic turnover within a community to determine the role of deterministic and stochastic processes. If βNTI values are >2 or <-2, the deterministic processes shape bacterial community assembly processes across all treatments. Stochastic processes may play a significant role in community assembly processes when the values of βNTI are between −2 and 2. The βNTI > 2 showed significantly more phylogenetic turnover than predicted, often interpreted by chance as variable selections, while βNTI values <2 referred to less phylogenetic turnover than expected, i.e., homogeneous selection (Stegen et al., 2013). The Bray–Curtis-based Raup–Crick metric (RC_bray_) was further determined as described by Stegen et al. (2013) to quantify the role of specific stochastic processes. In brief, the obtained values of RC_bray_ ranged between −1 and 1. We compared the βNTI values between 2 and−2 and RC_bray_ values to further determine stochastic assembly processes. Thus, when βNTI values are between 2 and −2 and RC_bray_ values > 0.95, the assembly process is dispersal limitation, when βNTI values are between 2 and −2 and RC_bray_ values are between 0.95 and −0.95, the process is drift, and when βNTI values are between 2 and −2 and RC_bray_ values <-0.95 is homogenizing dispersal (Liu et al., 2020).

### 2.6. Statistical and bioinformatics analysis

Quantitative insights into the microbial ecology (QIIME; http://qiime.org/) (Caporaso et al., 2012) workflow was used to obtain quality-filtered sequences and high-quality reads. The reads were compared with the reference database (http://drive5.com/uchime/uchime_download.html), using the UCHIME algorithm to find chimeric sequences and remove them to obtain clean and effective reads. Alpha diversity was calculated using MOTHUR software (http://www.mothur.org). For alpha diversity, Chao1 and ACE indices were used to estimate richness, while we used Shannon and Simpson indices to estimate diversity. For β-diversity, principal coordinate analysis (PCoA) scaling was used for the clustering of different samples (Li et al., 2014). Functional gene predictions were performed using the PICRUSt (Langille et al., 2013). Heatmap was generated using ggplot2 in RStudio software. All statistical analyses were completed using SPSS package release 22.0 (SPSS Inc., Chicago, USA). Significance was accepted at a *p*-value of <0.05 unless otherwise noted. The microbial community network was built by calculating correlations using co-occurrence network (CoNet) inference. Using an ensemble method, the three metrics—Pearson's and Spearman's correlations and Bray–Curtis dissimilarities between paired OTUs—were merged. Spearman's correlation between microbial taxa was considered statistically robust when Spearman's correlation coefficient (*r*) is >0.6 and the *p*-value is <0.01. We calculated the relative abundance of OTUs in all samples and then identified the OTUs that were dominant with a relative abundance >0.5% in all samples. These dominant OTUs with relative abundance >0.5% were selected for network construction. We selected the most abundant bacterial taxa belonging to these OTUs at the phyla level in all samples for comparative analysis. The *p*-values for the four measures were integrated using the Brownian methodology and then corrected using the Benjamini–Hochberg method to lessen the probability of false-positive results. The network was seen using Cytoscape software. Different microbial taxa were represented by the nodes in the microbiome network (OTUs). The network edges showed physiologically or chemically relevant relationships and represented pairwise correlations between nodes. Groups of closely related nodes comprise modules (i.e., groups of coexisting or co-evolving microbes) (Shannon et al., 2003; Banerjee et al., 2016). Mantel tests with Pearson's correlation coefficient and 999 permutations were performed to analyze the correlation between βNTI values and Euclidean distances in environmental parameters.

## 3. Results

### 3.1. Soil bacterial alpha diversity

The Illumina MiSeq sequencing produced 622,518 high-quality reads from four treatments. A total of 5,199 unique OTUs were identified from all the samples at 97% sequence similarity. Alpha diversity results of four treatments are shown in Table 1. The bacterial richness indices Chao1 and ACE showed higher richness in the CntWtS treatment, while lower bacterial richness was found in CntWnt. Shannon and Simpson diversity indices showed higher diversity in CntWt but lower diversity in the CntWntS treatment. The tillage with straw retention treatments increased soil bacterial richness, while no-tillage with straw retention decreased bacterial diversity. Therefore, no-tillage and straw retention practices significantly changed bacterial diversity.

**Table 1:** Soil bacterial richness and diversity in different treatments.

**Soil treatments**	**Reads**	OTUs^*^	**Coverage**	**Richness**		**Diversity**	
				**Chao**	ACE^*^	**Shannon**	**Simpson**
CntWtS	53,707	4,295	0.980	4,406 ± 177^a^	4,507 ± 208^a^	9.47 ± 0.02^b^	0.993 ± 0.00^ab^
CntWntS	52,949	4,117	0.979	4,325 ± 232^ab^	4,392 ± 269^ab^	9.29 ± 0.15^b^	0.992 ± 0.00^b^
CntWt	52,104	4,323	0.980	4,326 ± 114^ab^	4,366 ± 145^ab^	9.67 ± 0.09^a^	0.995 ± 0.00^a^
CntWnt	48,745	3,662	0.978	3,871 ± 385^b^	3,939 ± 409^b^	9.44 ± 0.06^b^	0.994 ± 0.00^ab^

OTU, operational taxonomic unit; ACE, abundance-based coverage estimator.

Different lowercase letters show significant differences at p < 0.05.

### 3.2. Soil bacterial β-diversity

**Figure 1:**
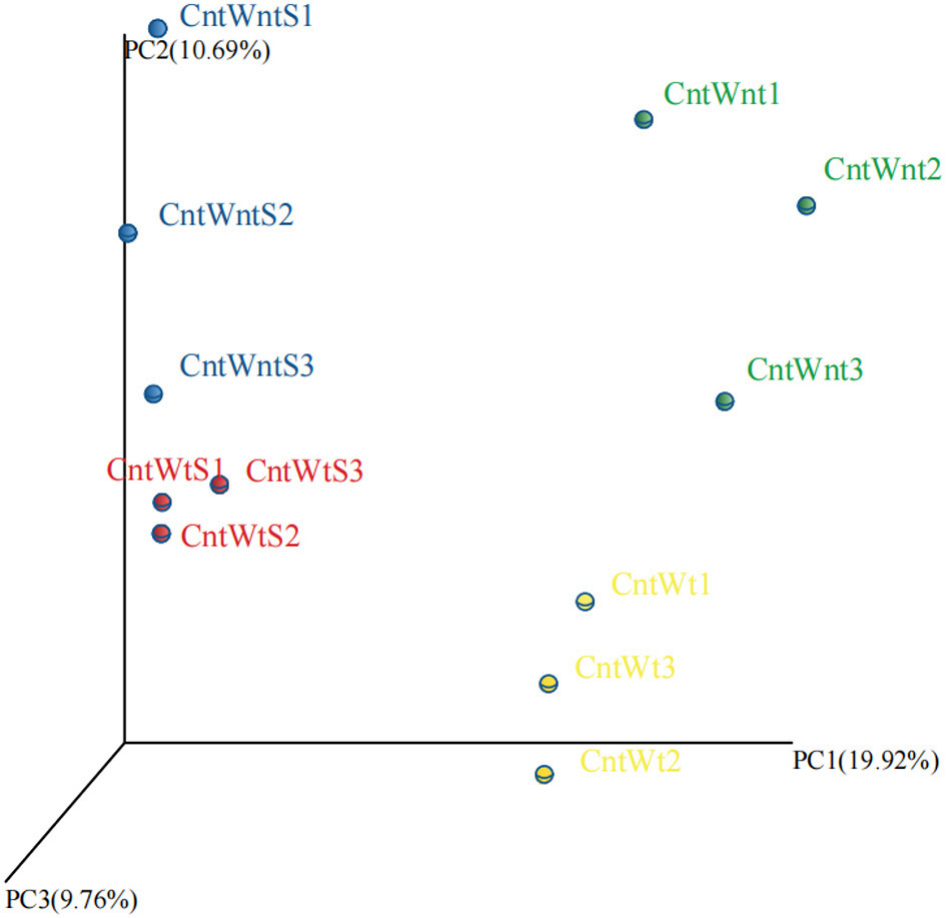
Principal Coordinates Analysis (PCoA) of unweighted UniFrac on 16S rRNA gene sequences from four treated soil samples with three replicates.

The PCoA based on the OTUs level is shown in Figure 1. PCoA extracts coordinate axes that reflect the difference between samples. Samples containing similar community composition cluster together at the PCoA plot. The CntWntS and CntWtS had less diverse communities than CntWnt and CntWt, indicating that straw retention has a stronger influence on the bacterial population than tillage practices.

### 3.3. Phylogenetic analysis of soil bacterial community

A total of 49 phyla were obtained from all samples using the MOTHUR program. The predominant phyla (≥1%) included *Proteobacteria, Acidobacteria, Planctomycetes, Firmicutes, Actinobacteria, Bacteroidetes, Chloroflexi, Gemmatimonadetes, Crenarchaeota, WS3, Nitrospirae, Verrucomicrobia*, and *OD1* in all treatments (Supplementary Figure S1). The relative abundance of *Proteobacteria* and *Bacteroidetes* was higher in CntWtS compared with CntWnt treatment. Similarly, the relative abundance of *Acidobacteria, Chloroflexi, Actinobacteria*, and *Verrucomicrobia* was much higher in CntWt and *Crenarchaeota*, and *OD1* and *WS3* were higher in CntWntS.

**Figure 2:**
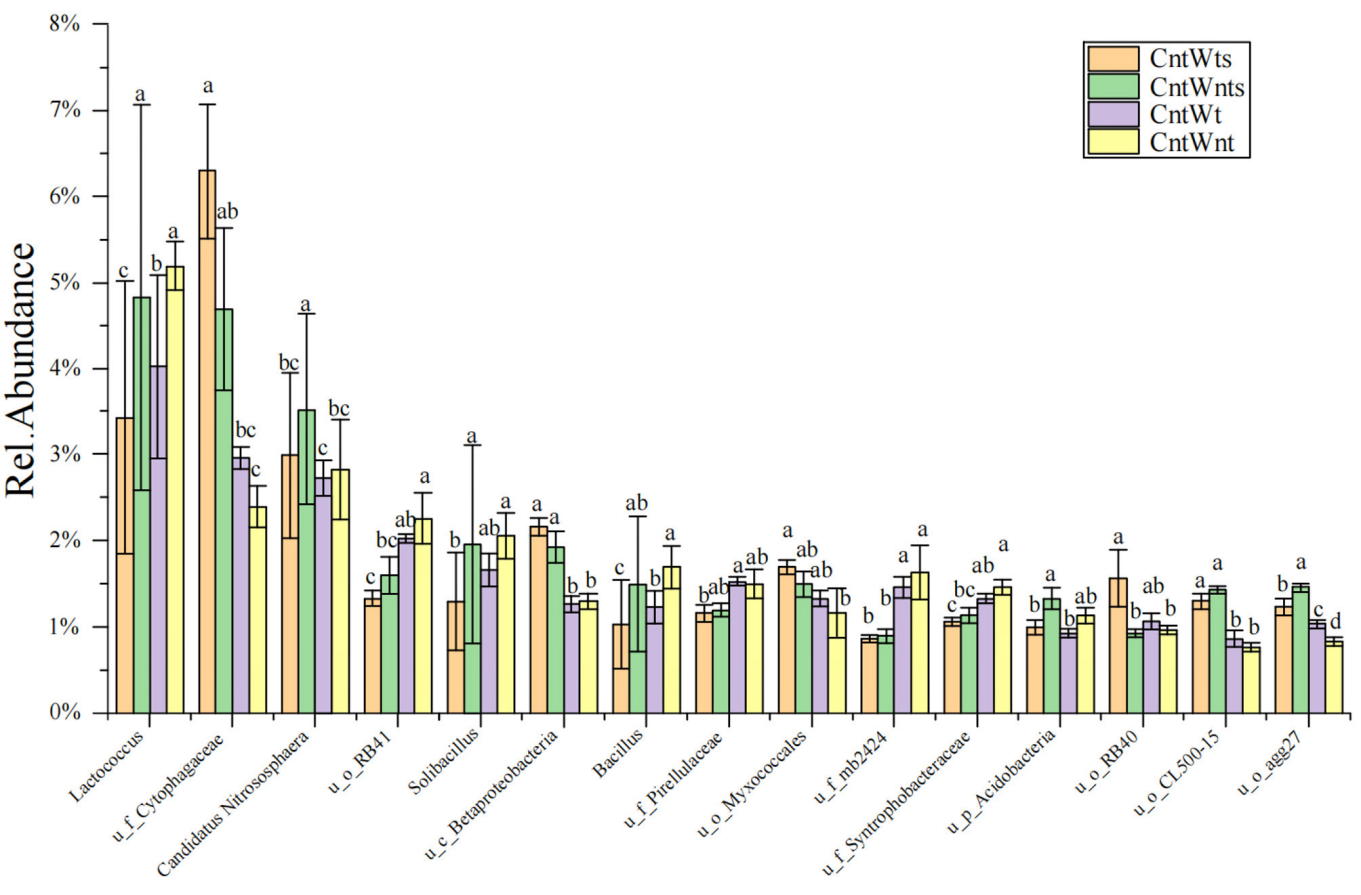
Relative abundances (≥ 1%) of dominant genera between four different treatments. “u” represents unclassified members at the genus level. Different lowercase letters show significant differences (*p* < 0.05).

The abundance of dominant (≥1%) classified and unclassified genera is shown in Supplementaary Table S1. In the CntWtS treatment, the relative abundance of unclassified genera *Cytophagaceae, Sinobacteraceae, MND, envOPS12, Betaproteobacteria, Myxococcales, RB40, Xanthomonadaceae*, and *Piscirickettsiaceae* was significantly higher than in other treatments. In the CntWntS treatment, the relative abundance of the classified genus *Candidatus Nitrososphaera* and the unclassified genera *Sediment-1, Acidobacteria, CL500-15, agg27*, and *ABY1* was significantly higher than in other treatments. The relative abundance of unclassified genera within the *Pirellulaceae, Rhodospirillaceae, Sva0725, Chitinophagaceae*, and *WD2101* was also higher in the CntWt treatment compared with treatments. Classified bacterial genera *Lactococcus, Solibacillus, and Bacillus* and unclassified genera *iii1-15, RB41, Gemm-1 0319-6A21, mb2424, Haliangiaceae, and Syntrophobacteraceae* had significantly higher abundance in the CntWnt treatment (Figure 2).

### 3.4. Phylogenetic β-diversity analysis of soil bacterial community assembly dynamic

**Figure 3:**
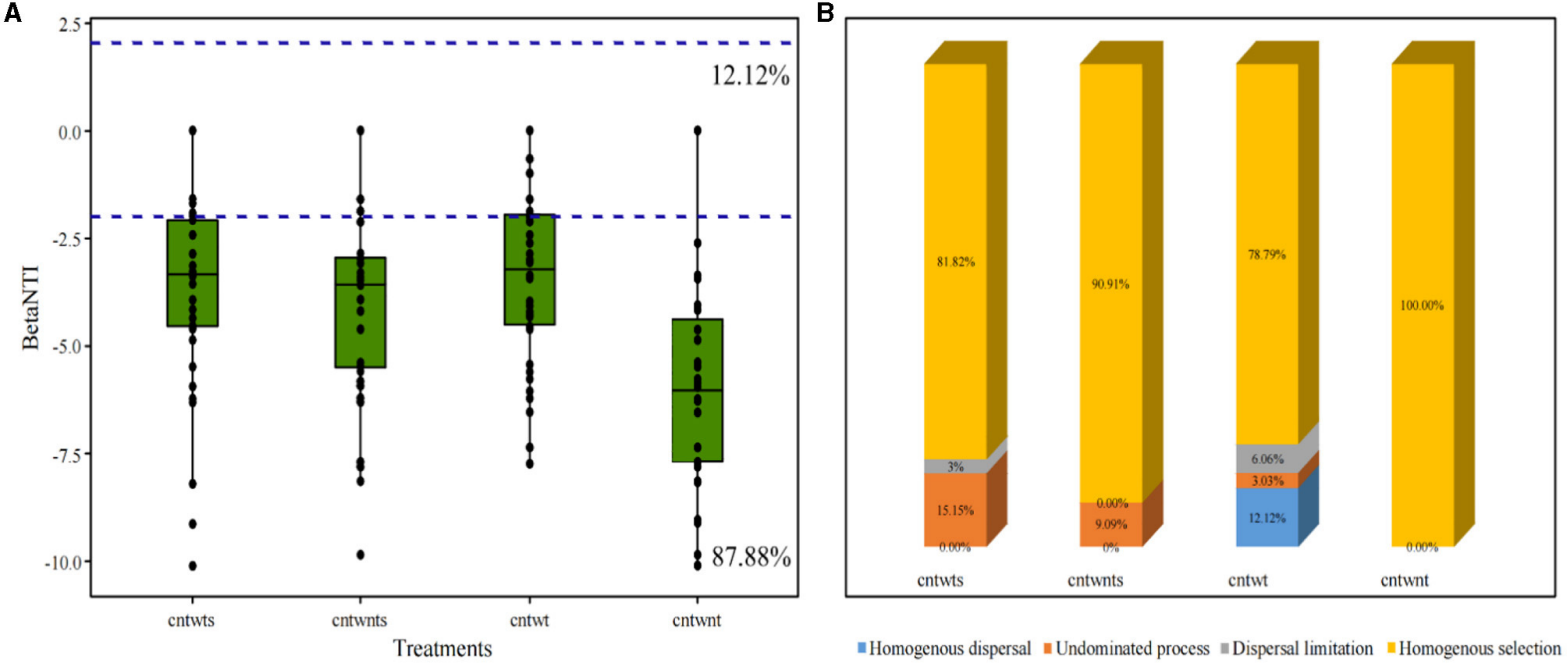
Boxplot of βNTI values grouped by treatment of the soil. **(A)** Horizontal blue dashed lines indicate lower and upper betaNTI significant thresholds (−2 and +2) respectively. **(B)** Percentage of bacterial community assembly governing processes.

We determined the β NTI for paired samples to assess whether neutral or niche-based mechanisms better explain the assembly of the bacterial community in different soil with different tillage treatments (Figure 3A). Our results indicated that deterministic processes assembled 87.88% of the bacterial community, and βNTI scores below −2 showed homogeneous selection as the dominant mechanism shaping community assemblage. In comparison, 12.12% community exhibited stochastic assembly processes and the βNTI values were below 2 and above −2. Quantitative assembly mechanism analysis further revealed that in CntWt treatments, homogeneous selection dominated assembly processes followed by homogeneous dispersal (Figure 3B). Interestingly, our study showed community assembly dynamic was not driven by variable selection in all treatments. The Mantel test was performed for βMNTD and βNTI values and measured environmental variables for further validations. Here, we applied both matrices and the results indicated that measured environmental variables and βMNTD and βNTI values were non-significantly correlated with community assembly processes (Supplementary Figure S3).

### 3.5. Bacterial community and environmental factor analysis

**Figure 4:**
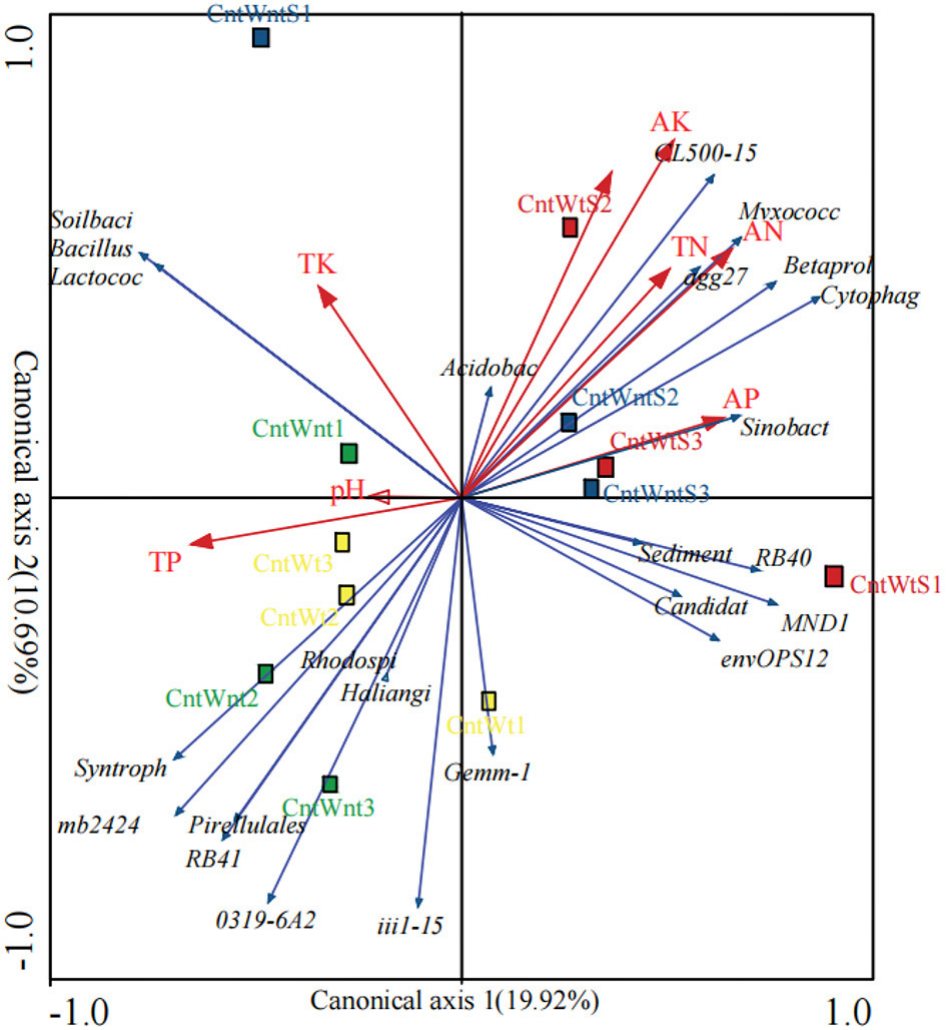
Redundancy analysis of bacterial communities in different residue and tillage treatment soils as affected by environmental properties (b). AP, Available Phosphorus; TP, Total Phosphorus; TK, Total Potassium; SOC, Soil Organic Carbon; AK, Available Potassium; AN, Available Nitrogen, TN: Total Nitrogen. The percentage indicates the proportion of variation elucidated by the first and second canonical axes.

The relationship between the dominant genera and environmental factors analyzed by the redundancy analysis (RDA) method (Figure 4) shows that bacterial community structure is affected by various environmental factors under conservation tillage treatments. The phylum *Proteobacteria* positively correlated with TN and VP and negatively correlated with TP. *Bacteroidetes* were positively correlated with AN, VP, and VK and negatively correlated with TP. *Actinobacteria* negatively correlated with AN and VK. The phyla WS3 positively correlated with TN and VK. *Nitrospirae* negatively correlated with OC and AN. OD1 was positively correlated with OC, TN, AN, and VK (Supplementary Table S4). Unclassified, such as *Sinobacteraceae, Betaproteobacteria, Myxococcales, Acidobacteria, Cytophagaceae*, and *Sediment-1*, positively correlated with total nitrogen. The available nitrogen concentration positively correlated with the abundance of *Betaproteobacteria, Cytophagaceae*, and *Sediment-1* and negatively correlated with the abundance of unclassified genera *RB41, mb2424*, and *031F9-6A21*. Unclassified *Acidobacteria* positively correlated with organic carbon. Classified genus *Lactococcus* and unclassified *Sinobacteraceae, MND1, Myxococcales*, and *Cytophagaceae* were positively associated with available phosphorus, while other *Betaproteobacteria* were negatively associated with total phosphorus. Soil-available potassium positively correlated with unclassified *Betaproteobacteria, Myxococcales, mb2424, Acidobacteria, Cytophagaceae*, and *Sediment-1* and was not correlated with total potassium. Soil pH was negatively correlated with classified *Lactococcus* and unclassified *Xanthomonadaceae* (Supplementary Table S3).

### 3.6. Bacterial community co-occurrence network

**Figure 5:**
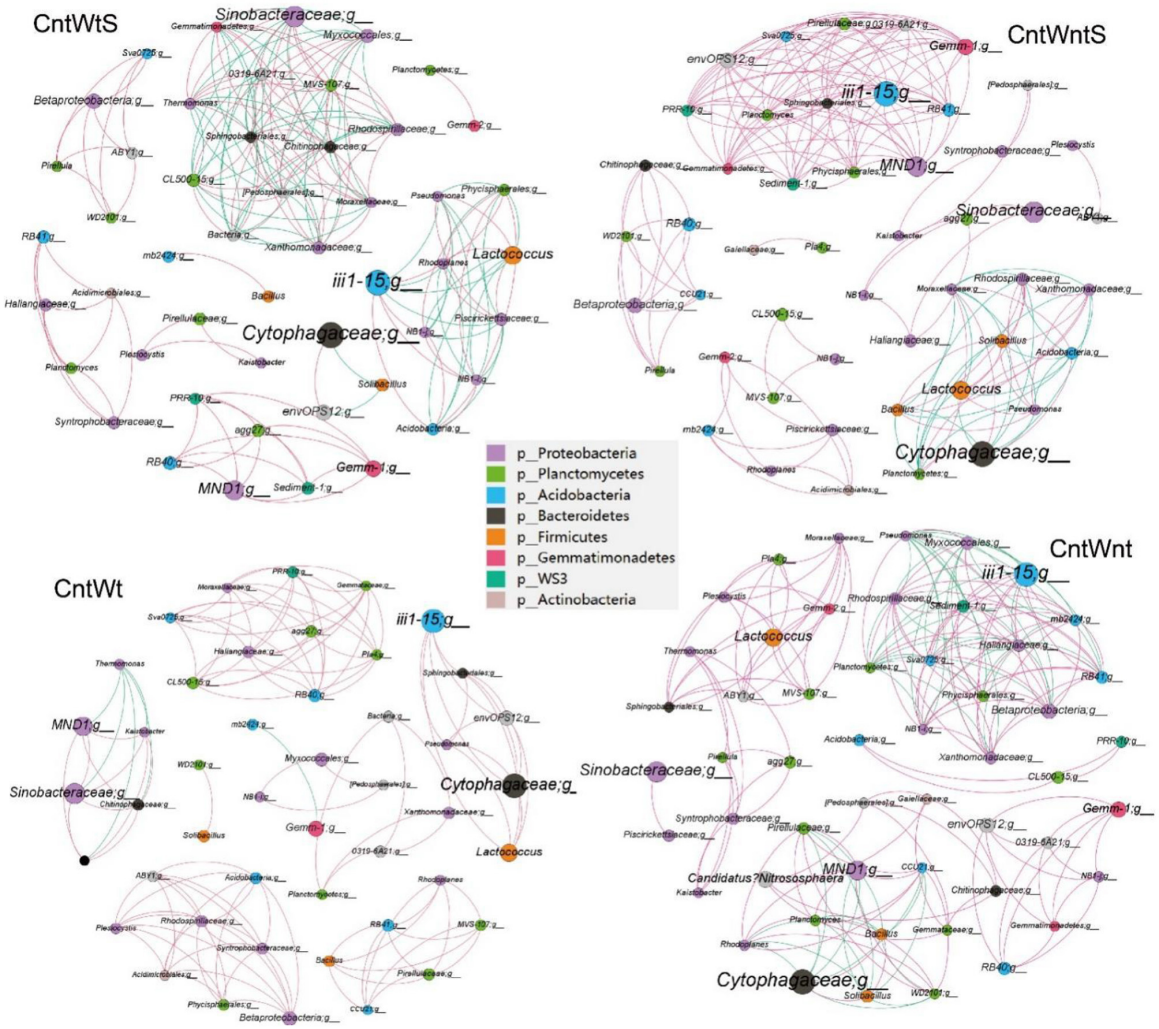
The bacterial co-occurrence networks under the CntWtS and CntWntS, CntWt and CntWnt treatments based on correlation analysis in the field experiment. A connection stands for a strong (Spearman's R > 0.9) and significant (*P* value < 0.01) correlation for different treatments. Each node name represents a taxon at the bacterial genus level, with unidentified genus names ending in; g_The size of each node is proportional to the number of links (degree), The networks in the field experiment are colored by the bacterial phyla. Each bacterial network consists of different closely related bacterial modules to identify keystone taxa (module hubs). The size of each node is proportional to the number of relative abundances. The red edges indicate positive interactions between two bacterial nodes, while green edges indicate negative interactions.

We constructed microbial co-occurrence networks based on the correlation analysis of taxonomic profiles in different tillage practices to further understand the impact of different tillage practices on microbial composition (Figure 5). Overall, 49, 50, 46, and 50 nodes were inter-linked 173 (57.80% positive and 42.2% negative), 185 (83.78% positive and 16.21% negative), 120 (95% positive and 5% negative), and 194 (70.10% positive and 29.90% negative) in CntWts, CntWntS, CntWt, and CntWnt, respectively (Table 2). The significant and robust associations between taxa (average degree) were higher in CntWnt than CntWntS, CntWtS, and CntWt (7.18, 7.14, 6.93, and 4.36, respectively). However, in CntWt and CntWntS, the positive connections were higher at 95 and 83.78%, respectively, while lower in CntWts at 75.80%, showing that the connections between OTUs were disturbed during different tillage practices. Interacting microbial networks clustered into modules can be examined to find significant modular relationships. Our results indicate that bacterial networks can all be broken down into smaller interconnected modules, with individual nodes exhibiting different roles in the microbial network. The bacterial taxa in the network consisted mainly of *p__Proteobacteria, p__Planctomycetes, p__Acidobacteria, p__Bacteroidetes, p__Firmicutes*, and *p__Gemmatimonadetes*. In the CntWtS network, *Sinobacteraceae;g*__(*p__Proteobacteria*) was detected as the most critical taxa (strongest interaction). In CntWntS network, iii1-15;g__(*p__Acidobacteria*) and *MND1*;g__ (*p__Proteobacteria*) were similarly designated as key taxa. The relatively few and unimportant key taxa in CntWt are *Cytophagaceae*;g__(*p__Bacteroidetes*). The key taxa in CntWnt are iii1-15;g__(*p__Acidobacteria*) and *Cytophagaceae*;g__(*p__Bacteroidetes*).

**Table 2:** Topological properties of a correlation network diagram of soil bacterial communities at the genus level in soils.

**Parameters**	**CntWtS**	**CntWntS**	**CntWt**	**CntWnt**
Number of nodes	49	50	46	50
Total links	173	185	120	194
Positive links	100	155	114	136
Negative links	73	30	6	58
Network density	0.141	0.149	0.116	0.136
Average degree	6.939	7.143	4.364	7.185
Modularity	0.653	0.651	0.805	0.713
No. of large modules	6	5	4	12

### 3.7. Functional predictions

**Figure 6:**
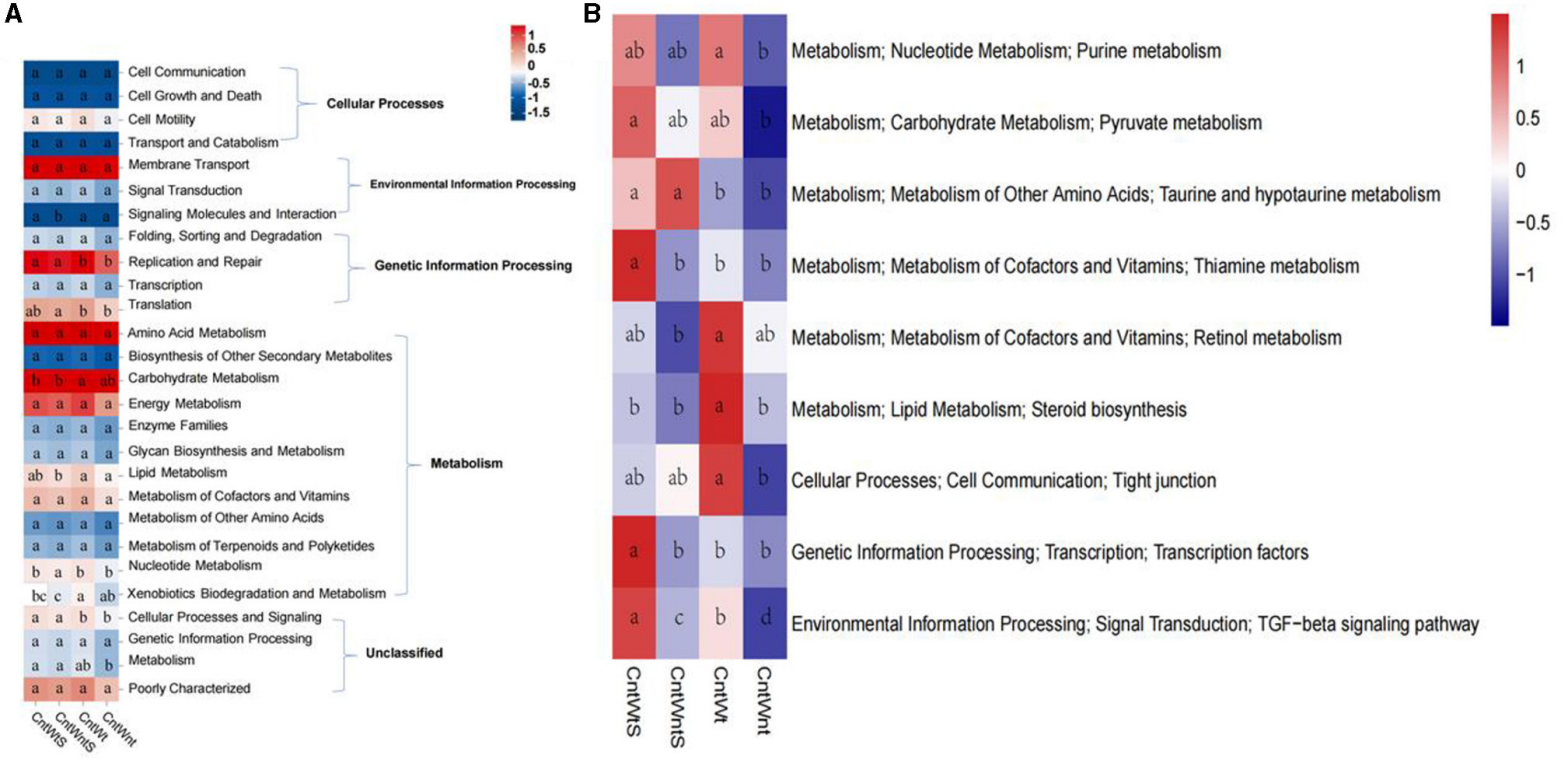
Predicted function of bacteria in four treatments. Values of each functional gene (row) was *z*-zero transformed. **(A)** KEGG level I and II. **(B)** Heatmap shows the third level of KEGG. The significant tests of gene distribution between groups was completed using ANOVA *p* < 0.05.

The functional prediction was carried out using the KEGG database. It was classified into seven major categories and 41 sub-categories (Figure 6A). Functional prediction indicated that metabolisms of xenobiotic biodegradation, nucleotide, lipids, energy, amino acids, and carbohydrate were significantly higher in tillage without straw retention treatment. At the same time, they were significantly lower in no-tillage and straw retention treatments. These findings suggest that tillage treatment significantly increases metabolic activities. Tillage with straw retention treatments showed significantly increased metabolism compared to no-tillage and straw retention treatment (Figure 6B). Our findings suggest that among all treatments, no-tillage and straw retention significantly decreased overall metabolic activity potential compared to control and other treatments.

## 4. Discussion

### 4.1. Effects of conservation tillage practices on soil bacterial richness and diversity

In our experiment, bacterial richness and diversity were lower in no-tillage treatments with or without crop straw retention. The no-tillage practices benefit the formation of soil micro-aggregates, which have different O_2_ concentrations inside and outside of the aggregates (Zhang X. et al., 2018). Some studies reported decreased bacterial diversity following organic material alteration, which helps the enrichment of copiotrophic taxa in soil (Fernandez, 2015; Lian et al., 2016). Our results indicate that tillage with straw retention treatment (CntWtS) increased bacterial richness and decreased bacterial diversity (Table 1), which is consistent with the previous study (Zhang Y. et al., 2018). While some studies reported decreased bacterial diversity following organic material amendment, ascribed to the incorporated material favoring the copiotrophic type microbial taxa (Ceja-Navarro et al., 2010; Fernandez, 2015). Soil disturbance caused by tillage and crop residues has been demonstrated as an important component affecting the microbial community (Jansa et al., 2002; Six et al., 2006; Cookson et al., 2008). A recent study showed that the soil aggregates and tillage practices affected soil bacterial communities compared with no-residue treatment (Zhang X. et al., 2018). The connection between tillage and soil bacterial communities may be complex, as tillage affects the soil's physiochemical properties and has a mutually dependent relationship with soil bacterial communities (Trivedi et al., 2017). Another study has shown that leaving crop residue on the soil surface increases soil OC, and crop residues with more cellulose or lignin and less N will decompose more slowly (Benbi and Khosa, 2014; Panettieri et al., 2015). The deposition of N significantly affected the relative abundances of bacterial phyla but reduced the bacterial diversity and changed the bacterial composition at low-level taxonomic levels (Frey et al., 2014; Freedman and Zak, 2015). According to Jiao et al. (2004), tillage practices affect the supply of soil nutrients, which include soil moisture content, OM decomposition, and porosity of the soil.

### 4.2. Inferring of conservation tillage practices on microbial community assembly processes

**Figure 7:**
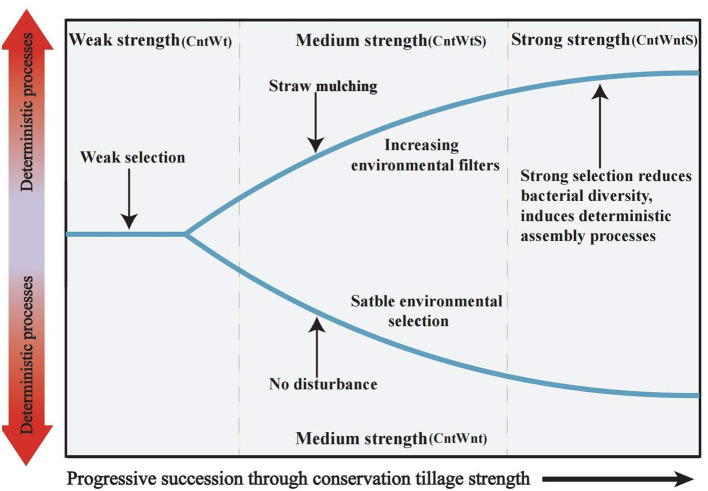
Conceptual model showing how ecological selection determines microbial community assembly processes by different tillage treatments. Weak strength in samples with tillage and no straw retention (CntWt); Medium strength in samples of no-tillage with no straw retention (CntWnt) and tillage with straw retention (CntWtS); Strong strength in samples with no-tillage with straw retention (CntWntS).

Several studies on microbial community ecology focus on the relative contribution of deterministic and stochastic processes (Stegen et al., 2013; Zhang et al., 2016; Zhou and Ning, 2017). In this study, the dominance of homogeneous selection in community assembly processes was confirmed by quantifying various ecological mechanisms in community assembly (Figure 3). Bacterial community assembly processes showed different trends, such as higher stochastic processes (15.15% undominated and 3% dispersal limitation) associated with tillage practices with no-straw retention treatments. On the other hand, no-tillage with straw retention was more associated with deterministic homogeneous selection. Finally, no-tillage with no-straw retention showed 100% homogeneous selection. To better understand microbial community assembly processes, we have established a conceptual model (Figure 7). This shows that ecological processes can occur in the following forms: (i) under weak selection (CntWt), microbial community assembly processes show the contribution of stochastic processes; (ii) under medium selection (CntWts), environmental selection leads to deterministic processes dominating the microbial communities; and (iii) under strong selection (CntWts), microbial diversity is reduced, which ultimately induces the deterministic processes. Our findings are contrary to previous studies indicating that tillage practices impact bacterial assembly processes (Wang et al., 2020; Li et al., 2021), while in line with Zhuxiu et al. (2021), who found that both stochastic and deterministic processes play a significant role in bacterial community assembly processes under conservation tillage practices.

### 4.3. Effects of conservation tillage practices on microbial community structure

The 16S rRNA sequencing indicated that at the phylum level, bacterial community composition was very similar in all treatments, dominated by *Proteobacteria, Acidobacteria, Planctomycetes, Firmicutes, Bacteroidetes, Chloroflexi, Gemmatimonadetes, Actinobacteria, Nitrospirae*, and *Crenarchaeota* (Supplementary Figure S1). This result supports several other studies (Navarro-Noya et al., 2013; Wang et al., 2016). The relative abundance of *Proteobacteria* and *Bacteroidetes* significantly increased in tillage with straw retention treatments, which are responsible for labile carbon sources and increased in crop residue retention treatment (Fierer et al., 2007; Navarro-Noya et al., 2013). Many studies revealed that an application of crop residue retention supported *Proteobacteria*, particularly for bacteria at order levels of this phylum, such as *Myxococcales, Pseudomonadales*, and *Sphingomonadales* (Wallenstein et al., 2007; de Gannes et al., 2013; De la Cruz-Barrón et al., 2017; Su et al., 2017). The relative abundance of *Betaproteobacteria* and *Myxococcales* was significantly higher in crop straw retention treatments (Figure 2), *Myxococcales* are well-known to be able to decompose macromolecules, and some members can decompose cellulose (Bernard et al., 2007; Ceja-Navarro et al., 2010; Negassa et al., 2015). A previous study showed that *Betaproteobacteria* growth was positively linked with the soil's quality and amount of nutrients (Lin et al., 2010). Tillage alters the surrounding environment for *Pseudomonas, Rudaea, Bacillus*, and other bacteria and then changes the decay process of residue (Xia et al., 2019). The relative abundance of the family *Cytophagaceae* was significantly higher in straw retention treatments but was not significantly influenced by either depth or tillage treatments (McBride et al., 2014; Cania et al., 2019). Members of *Cytophagaceae* are aerobic and cellulolytic, which help to regulate the carbon cycle (Monserrate et al., 2001).

Applying crop residues into the soil leads to a shift in the relative abundances of various phyla, favoring the prevalence of syntrophic microorganisms such as *Actinobacteria* and *Firmicutes* (Ramirez-Villanueva et al., 2015). Another study stated that tillage with crop rotation increased the relative abundance of *Acidobacteria* than in a no-tillage (Yin et al., 2010). However, in this study, the relative abundance of phyla *Acidobacteria* and *Firmicutes* decreased with the application of straw retention in tillage and no-tillage treatment (Supplementary Figure S1). The reduction in *acidobacteria* might be due to the high level of available carbon sources as it is reported that the relative abundance of *acidobacteria* was higher in the low level of carbon (Fierer et al., 2007), while in this study, total organic carbon was relatively higher with straw retention application (Zhang et al., 2022). The relative abundance of the genus *Lactococcus, Bacillus*, and *Solibacillus* (*Firmicutes*) and unclassified _o_*iii1-15* (*Actinobacteria*) decreased in tillage with straw retention treatment (Figure 2). Our findings are contrary to previous studies reported that the relative abundances of the plowed and no-tilled communities underwent significant changes during the growth stage, albeit with distinct trends. *Actinobacteria* are typically characterized as bacteria that thrive in nutrient-rich environments. These contrary findings might be due to experimental design, sampling size (Xue et al., 2021), soil types (Bickel and Or, 2020), and availability of nutrients (Bastida et al., 2021), which are predominant factors affecting microbial distribution. For example, a previous study showed that *Actinobacteria* appeared in oligotrophic conditions with low microbial activity (Jenkins et al., 2010; Che et al., 2016). Previous research also showed that *Lactococcus* represented 2.72% of the soil bacterial community and that the relative abundance of *Bacillus, Lactococcus, Streptococcus*, and *Enterococcus* in farmland, grassland, and woodland is significantly higher than in cropland (Cheng et al., 2018; Liang et al., 2019). This study also showed that the relative abundance of members of the phylum *Crenarchaeota* and class *Thermoplasmata* was higher in the 10–30 cm layer under conventional tillage with straw retention conditions, while tillage without straw retention increased the abundance of phylum Crenarchaeota's genus *Candidatus Nitrososphaera* (Barber et al., 1996). The significant correlation of phylum *Planctomycetes* with biomass and various pools of soil was noted, which is accountable for the anaerobic oxidation of ammonium (Buckley et al., 2006; Kuenen, 2008; Humbert et al., 2010).

However, the PCoA based on the unweighted UniFrac distance matrixes showed that straw retention and tillage system significantly affected the bacterial communities more than the control. These findings are in agreement with previous studies that reported that tillage practices and straw retention impact soil microbial community composition (Wang et al., 2017; Lu et al., 2019; Fernandez-Gnecco et al., 2022). The RDA showed that changes related to the different treatment and environmental factors were generated in bacterial communities (Negassa et al., 2015; de Graaff et al., 2019). To some extent, the bacterial community structure was affected by various environmental factors. TP and AK have a more significant impact on the bacterial community in treatments such as straw retention and tillage treatment (Figure 4).

### 4.4. Effects of conservation tillage practices on functions and bacterial network

In tillage with straw retention treatment, part of the metabolism (i.e., nucleotide, carbohydrate, and amino acid metabolism) significantly increased. Many studies have revealed that carbohydrate metabolism is an enzymatic process that changes external substrates into metabolic precursors, such as hydrolyzed polymers into monomers and d-fructose-6-phosphate and acetyl-CoA and pyruvate (Bräsen et al., 2014). Enzymes such as alpha-aminoadipic semialdehyde synthase and S-adenosylmethionine synthetase are involved in amino acid degradation (Guo et al., 2015; Gao et al., 2016). Yang et al. (2014) found that anaerobic microbes are involved in carbohydrate decay and energy conversion. In the amino acid metabolism which is a central process adopted by soil microbial communities in the presence of constant moisture stress is higher than normal environmental conditions (Fierer et al., 2012; Monard et al., 2012). The co-occurrence network further exhibited that total links are lower in no-tillage with straw retention and higher in tillage treatment, indicating that straw retention has a different effect on bacterial relationship. Tillage with no-straw retention impacts bacteria and demonstrates that no-tillage can make the network of soil biota more compact (Chaffron et al., 2010). No-tillage and straw retention can significantly promote the positive connection of dominant bacterial networks. The more compact network topology may be caused by several factors, which may be related to the no-tillage reduction in human microbial disturbances, and the return of straw to the bacteria to bring enough nitrogen and carbon sources, as well as appropriate habitat and breeding environment (Zaneveld et al., 2010). Another study reported that most studies consider the soil biota to be a black box, and little is known about the entire soil network (Schloss et al., 2009). In general, crop type (Fyles et al., 1988; Kaiser and Heinemeyer, 1993; Grayston et al., 1998), crop rotation (McGill et al., 1986; Dick, 1992), soil type (Girvan et al., 2003), sampling season (Spedding et al., 2004; Zhang et al., 2012), and agriculture management drive bacterial distributions in soils. In our study, straw retention treatments decreased soil bacterial diversity, and no-tillage treatments reduced the number of OTUs and bacterial diversity compared to tillage treatments, which differ from previous work in several significant ways. The contrary findings might be due to crop rotation because the wheat and maize straw return mode significantly changed bacterial diversity in soil and showed a downward trend (Zhang et al., 2023). Another reason might be an increase in risk disease pathogens, which may relate to a change in soil electrical conductivity and impact on soil microbial diversity, as reported in a previous study that maize straw reduces fungal diversity and fungal pathogens were abundant in maize straw-treated soil (Su et al., 2020).

## 5. Conclusion

We observed clear variations in soil physicochemical properties and microbial community structure by different tillage treatments. Different tillage practices significantly influenced soil properties except for pH and total potassium, while straw retention treatments significantly alter soil properties. Bacterial communities responded differently to different tillage treatments such as the relative abundance of the phyla *Proteobacteria, Bacteroidetes, WS3, OD1, Acidobacteria, Actinobacteria, Nitrospirae, Verrucomicrobia, Cytophagaceae*, and *Betaproteobacteria* was significantly affected by the addition of straw retention in tillage and no-tillage treatments. On the other hand, tillage practices with or without straw retention also changed some bacterial relative abundance, such as *Planctomycetes, Firmicutes, Gemmatimonadetes, Crenarchaeota, Chloroflexi*, and *Lactococcus*. Tillage without straw retention significantly affected soil bacterial diversity as compared to other treatments. Based on bacterial network analysis, no-tillage treatment can increase the co-occurrence between dominant bacterial genera. The relationship between bacterial genera with straw retention treatment was found to have different effects on tillage and no-tillage treatments. The function prediction analysis indicated that tillage treatment significantly increased the potential of metabolic activities. We can conclude that tillage practices and straw retention treatments are essential in soil microbial community structure and microbial metabolic activities. Current results revealed that we can alter microbial community structure in agricultural soil to improve soil fertility by managing tillage practices. We conducted this study in a controlled environment, and further research is needed on agricultural soils and open fields to determine the effect of long-term conservation tillage practices on soil bacterial community structure, functions, and their dynamics. Further experiments must consider testing different crop rotation and their impact on soil microbial community composition at a larger scale.

## Data availability statement

The original contributions presented in the study are included in the article/Supplementary material, further inquiries can be directed to the corresponding authors.

## Author contributions

HC and SH: conceptualization, supervision, and project administration. MuzK: methodology, formal analysis, resources, and writing—original draft preparation. HL: software. HC, SH, and MuzK: validation. AZ: investigation. SH: data curation. HL and MudK: writing—review and editing. HC: visualization and funding acquisition. All authors have read and agreed to the published version of the manuscript.
