# Supplementary material for: Conservation tillage practices affect soil microbial diversity and composition in experimental fields
Source: Front Microbiol. 2023 Aug 2;14:1227297. doi: 10.3389/fmicb.2023.1227297 (PMC10433396; doi:10.3389/fmicb.2023.1227297)
Supplement: Supplementary file 1 [file Data_Sheet_1.docx]

Conservation tillage practices affect soil microbial diversity and composition in experimental fields

Muzammil Hassan Khan^1^, Hao Liu^1^, Anning Zhu ^3^, Mudassir Hassan Khan^4^, Sarfraz Hussain ^1,2^* and Hui Cao ^1^*

^1^ College of Life Sciences/Key Laboratory of Agricultural Environmental Microbiology, Ministry of Agriculture, Nanjing Agricultural University, Nanjing 210095, China

^2^ Key Laboratory of Integrated Regulation and Resource Development on Shallow Lakes of Ministry of Education, College of Environment, Hohai University, Nanjing, 210098, China.

^3^ Fengqiu Agro-Ecological Experimental Station, State Key Laboratory of Soil and Sustainable Agriculture, Institute of Soil Science, Chinese Academy of Sciences, Nanjing 210008, China

Sciences Department, Karakoram International University Gilgit-Baltistan, Pakistan

***** Correspondence: Cao Hui: [hcao@njau.edu.cn, Sarfraz](mailto:hcao@njau.edu.cn,%20Sarfraz) Hussain: [20210936@hhu.edu.cn](mailto:20210936@hhu.edu.cn),

**Table S1** The significant differences between genera among differently treated soil samples.

| Genus | CntWtS (%) | CntWntS (%) | CntWt (%) | CntWnt (%) |
| --- | --- | --- | --- | --- |
| unclassified _o_iii1-15 | 6.00 ± 0.50^a^ | 4.93 ± 0.50^a^ | 6.27 ± 0.12^a^ | 6.70 ± 0.94^a^ |
| *Lactococcus* | 3.43 ± 1.58^a^ | 4.83 ± 2.24^a^ | 4.03 ± 1.07^a^ | 5.20 ± 0.28^a^ |
| unclassified_f_ Cytophagaceae | 6.30 ± 0.78^a^ | 4.70 ± 0.94^ab^ | 2.97 ± 0.12^bc^ | 2.40 ± 0.24^c^ |
| unclassified_f_ Sinobacteraceae | 4.60 ± 0.36^a^ | 4.20 ± 0.14^a^ | 4. 33 ± 0.12^a^ | 3.27 ± 0.09^b^ |
| unclassified_o_ MND1 | 4.03 ± 0.38^a^ | 3.60 ± 0.36^a^ | 3.57 ± 0.17^a^ | 3.27 ± 0.26^a^ |
| *Candidatus Nitrososphaera* | 3.00 ± 0.96^a^ | 3.53 ± 1.11^a^ | 2.73 ± 0.21^a^ | 2.83 ± 0.58^a^ |
| unclassified_c_ Gemm-1 | 2.63 ± 0.24^a^ | 2.90 ± 0.37^a^ | 2.53 ± 0.12^a^ | 3.10 ± 0.14^a^ |
| unclassified_o_ envOPS12 | 2.30 ± 0.24^a^ | 2.27 ± 0.34^a^ | 2.20 ± 0.08^a^ | 2.00 ± 0.28^a^ |
| unclassified_o_ RB41 | 1.33 ± 0.09^c^ | 1.60 ± 0.22^bc^ | 2.03 ± 0.05^ab^ | 2.27 ± 0.29^a^ |
| *Solibacillus* | 1.30 ± 0.57^a^ | 1.97 ± 1.16^a^ | 1.67 ± 0.19^a^ | 2.07 ± 0.26^a^ |
| unclassified_c_ Betaproteobacteria | 2.17 ± 0.09^a^ | 1.93 ± 0.19^a^ | 1.27 ± 0.09^b^ | 1.30 ± 0.08^b^ |
| *Bacillus* | 1.03 ± 0.52^a^ | 1.50 ± 0.78^a^ | 1.23 ± 0.19^a^ | 1.70 ± 0.24^a^ |
| unclassified_f_ Pirellulaceae | 1.17 ± 0.09^b^ | 1.20 ± 0.08^ab^ | 1.53 ± 0.05^a^ | 1.50 ± 0.16^ab^ |
| unclassified_o_ Myxococcales | 1.70 ± 0.08^a^ | 1.50 ± 0.00^ab^ | 1.33 ± 0.09^ab^ | 1.17 ± 0.29^b^ |
| unclassified_f_ 0319-6A21 | 1.13 ± 0.17^a^ | 1.10 ± 0.08^a^ | 1.57 ± 0.05^a^ | 1.60 ± 0.28^a^ |
| unclassified_f_ mb2424 | 0.87 ± 0.05^b^ | 0.90 ± 0.08^b^ | 1.47 ± 0.12^a^ | 1.63 ± 0.31^a^ |
| unclassified_f_ Rhodospirillaceae | 1.23 ± 0.12^a^ | 1.10 ± 0.08^a^ | 1.27 ± 0.09^a^ | 1.23 ± 0.12^a^ |
| unclassified_f_ Haliangiaceae | 1.13 ± 0.09^a^ | 1.07 ± 0.05^a^ | 1.27 ± 0.0^9a^ | 1.40 ± 0.24^a^ |
| unclassified_f_Syntrophobacteraceae | 1.07 ± 0.05^c^ | 1.13 ± 0.09^bc^ | 1.33 ± 0.05^ab^ | 1.47 ± 0.09^a^ |
| unclassified_o_ Sediment-1 | 1.20 ± 0.14^a^ | 1.37 ± 0.21^a^ | 1.00 ± 0.00^a^ | 1.17 ± 0.12^a^ |
| unclassified_p_ Acidobacteria | 1.00 ± 0.08^b^ | 1.33 ± 0.12^a^ | 0.93 ± 0.05^b^ | 1.13 ± 0.09^ab^ |
| unclassified_o_ RB40 | 1.57 ± 0.33^a^ | 0.93 ± 0.05^b^ | 1.07 ± 0.09^ab^ | 0.97 ± 0.05^b^ |
| unclassified_o_ CL500-15 | 1.30 ± 0.08^a^ | 1.43 ± 0.05^a^ | 0.87 ± 0.09^b^ | 0.77 ± 0.05^b^ |
| unclassified_o_ agg27 | 1.23 ± 0.09^b^ | 1.47 ± 0.05^a^ | 1.03 ± 0.05^c^ | 0.83 ± 0.05^d^ |
| unclassified_c_ Pla4 | 0.80 ± 0.00^bc^ | 0.97 ± 0.5^ab^ | 0.77 ± 0.09^c^ | 1.03 ± 0.05^a^ |
| unclassified_f_ Xanthomonadaceae | 1.20 ± 0.22^a^ | 0.73 ± 0.12^b^ | 0.93 ± 0.09^ab^ | 0.80 ± 0.08^ab^ |
| unclassified_o_ Sva0725 | 0.57 ± 0.09^b^ | 0.70 ± 0.08^ab^ | 1.03 ± 0.05^a^ | 0.97 ± 0.21^a^ |
| unclassified_f_ Chitinophagaceae | 0.93 ± 0.17^a^ | 0.57 ± 0.09^b^ | 1.00 ± 0.08^a^ | 0.70 ± 0.08^ab^ |
| unclassified_o_ WD2101 | 0.67 ± 0.09^b^ | 0.63 ± 0.05^b^ | 1.07 ± 0.05^a^ | 0.97 ± 0.21^ab^ |
| unclassified_c_ ABY1 | 0.87 ± 0.09^a^ | 1.03 ± 0.05^a^ | 0.57 ± 0.09^b^ | 0.27 ± 0.09^c^ |

Values in a column for each class superscript with different letters are significantly different from each other at p<0.05.

**Table S2** Topological parameters of bacterial genera network analysis.

| Parameters | Treatment name | | | |
| --- | --- | --- | --- | --- |
|  | CntWtS | CntWntS | CntWt | CntWnt |
| Clustering coefficient | 0.451 | 0.902 | 0.913 | 0.943 |
| Connected component | 10 | 10 | 9 | 9 |
| Shortest path | 173 | 370 | 240 | 388 |
| Average number of neighbors | 6.784 | 7.255 | 5.217 | 7.321 |
| Number of nodes | 51 | 51 | 46 | 53 |
| Total links | 173 | 185 | 120 | 194 |
| Positive links | 102 | 155 | 114 | 136 |
| Negative links | 71 | 30 | 6 | 58 |

**Table S3** Pearson correlation (r) between the relative abundances (≥ 1%) of dominant genera and soil characteristics； N=12

| Phyla | Taxon (classified and unclassified) | OC | TN | AN | TP | AP | TK | AK | pH |
| --- | --- | --- | --- | --- | --- | --- | --- | --- | --- |
| Proteobacteria | unclassified_f_Sinobacteraceae | .238 | **.619^*^** | .329 | **-.664^*^** | **.580^*^** | -.148 | .485 | -.512 |
|  | unclassified_o_MND1 | .132 | .553 | .341 | **-.614^*^** | **.593^*^** | -.318 | .424 | -.531 |
|  | unclassified_c_Betaproteobacteria | .496 | **.747^**^** | **.779^**^** | -.516 | **.630^*^** | -.133 | **.853^**^** | -.293 |
|  | unclassified_o_Myxococcales | .507 | **.620^*^** | .477 | **-.581^*^** | **.769^**^** | .044 | **.659^*^** | -.328 |
|  | unclassified_f_Rhodospirillaceae | -.059 | .400 | .038 | -.457 | .452 | -.055 | .190 | -.573 |
|  | unclassified_f_Haliangiaceae | -.059 | -.092 | -.326 | -.176 | .444 | -.165 | -.182 | -.149 |
|  | unclassified_f_Syntrophobacteraceae | -.291 | -.145 | -.443 | .097 | .190 | -.085 | -.223 | -.076 |
|  | unclassified_f_Xanthomonadaceae | -.046 | .400 | .228 | **-.774^**^** | .544 | -.257 | .271 | **-.715^**^** |
| Acidobacteria | unclassified _o iii1-15 | -.488 | -.036 | -.249 | -.372 | .358 | -.489 | -.171 | -.385 |
|  | unclassified_o_RB41 | -.566 | -.444 | **-.675^*^** | .307 | -.146 | -.259 | -.533 | .195 |
|  | unclassified_f_mb2424 | **-.730^**^** | -.540 | **-.790^**^** | .211 | -.187 | -.253 | **-.652^*^** | -.010 |
|  | unclassified_p_Acidobacteria | **.683^*^** | **.631^*^** | .551 | .168 | .155 | .099 | **.680^*^** | .064 |
|  | unclassified_o_RB40 | .098 | .537 | .425 | **-.803^**^** | **.656^*^** | -.313 | .370 | **-.662^*^** |
| Firmicutes | *Lactococcus* | .106 | .035 | -.110 | .251 | -.138 | .334 | .217 | .079 |
|  | *Solibacillus* | .203 | .155 | -.082 | .265 | -.111 | .540 | .208 | .046 |
|  | *Bacillus* | .185 | .058 | -.102 | .289 | -.168 | .453 | .187 | .024 |
| Bacteroidetes | unclassified_f_Cytophagaceae | .501 | **.622^*^** | **.764^**^** | **-.686^*^** | **.687^*^** | -.145 | **.800^**^** | -.250 |
| Crenarchaeota | *Candidatus nitrososphaera,* | .252 | .217 | .282 | -.070 | .377 | -.402 | .315 | .146 |
| Planctomycetes | unclassified_f_Pirellulaceae | -.447 | -.221 | -.497 | -.071 | .155 | -.341 | -.257 | -.071 |
| Chloroflexi | unclassified_o_envOPS12 | .005 | .326 | .223 | -.440 | .537 | -.418 | .413 | -.062 |
| VS3 | unclassified_o_Sediment-1 | .367 | **.614^*^** | **.583^*^** | -.086 | .252 | -.300 | **.615^*^** | -.138 |
| Nitrospirae | unclassified_f_0319-6A21 | **-.598^*^** | -.263 | **-.594^*^** | .028 | -.035 | -.333 | -.511 | -.234 |
| Gemmatimonadetes | unclassified_c_Gemm-1 | .159 | .389 | .251 | .041 | .266 | -.295 | .307 | -.122 |

Abbreviations: OC, organic carbon; TN, total nitrogen; AN, available nitrogen; TP, total phosphorus; VP, available phosphorus; TK, total potassium; VK, available potassium; pH, power of hydrogen; ace, abundance-based coverage estimator. (**) The correlation (p<0.01, bilateral) significant, (*) The correlation (p<0.05, bilateral) significant.

| Taxonomy | Soil Properties | | | | | | | |
| --- | --- | --- | --- | --- | --- | --- | --- | --- |
|  | OC | TN | AN | TP | VP | TK | VK | pH |
| Proteobacteria | 0.303 | **.630^*^** | 0.369 | **-.580^*^** | **.644^*^** | -0.054 | 0.549 | -0.5 |
| Acidobacteria | -0.49 | -0.119 | -0.409 | -0.169 | 0.229 | -0.391 | -0.277 | -0.242 |
| Planctomycetes | 0.196 | 0.422 | 0.147 | -0.191 | 0.443 | -0.163 | 0.383 | -0.15 |
| Firmicutes | 0.242 | 0.109 | -0.055 | 0.321 | -0.176 | 0.463 | 0.242 | 0.092 |
| Bacteroidetes | 0.417 | 0.559 | **.623^*^** | **-.730^**^** | **.749^**^** | -0.144 | **.727^**^** | -0.261 |
| Chloroflexi | -0.119 | 0.181 | -0.027 | -0.421 | 0.555 | -0.367 | 0.186 | -0.182 |
| Gemmatimonadetes | 0.076 | 0.393 | 0.212 | -0.192 | 0.418 | -0.314 | 0.341 | -0.235 |
| Actinobacteria | -0.42 | -0.353 | **-.748^**^** | -0.088 | 0.092 | 0.017 | **-.592^*^** | -0.195 |
| Crenarchaeota | 0.257 | 0.224 | 0.289 | -0.079 | 0.385 | -0.4 | 0.326 | 0.141 |
| WS3 | 0.428 | **.664^*^** | 0.557 | -0.095 | 0.308 | -0.212 | **.614^*^** | -0.163 |
| Nitrospirae | **-.590^*^** | -0.333 | **-.620^*^** | 0.11 | -0.061 | -0.318 | -0.521 | -0.182 |
| Verrucomicrobia | -0.067 | 0.004 | -0.228 | -0.387 | 0.559 | -0.208 | 0.061 | -0.082 |
| OD1 | **.668^*^** | **.770^**^** | **.651^*^** | -0.417 | 0.508 | 0.071 | **.791^**^** | -0.225 |

**Table S4** Pearson correlation (r) between the relative abundances (≥ 1%) of dominant phyla and soil characteristics； N=12

Abbreviations: OC, organic carbon; TN, total nitrogen; AN, available nitrogen; TP, total phosphorus; VP, available phosphorus; TK, total potassium; VK, available potassium; pH, power of hydrogen; ace, abundance-based coverage estimator. (**) The correlation (p<0.01, bilateral) significant, (*) The correlation (p<0.05, bilateral) significant.


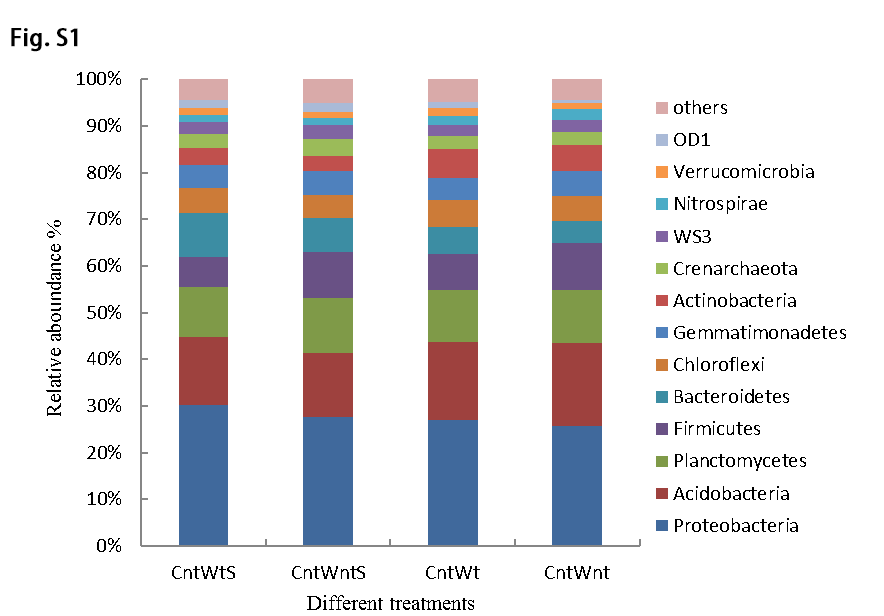


**Figure S1** Relative abundance of different bacterial phyla. The values on the Y-axis show relative abundance (%) of phyla, and the X-axis shows different soil treatments. Sequences that could not be classified into any known group were labeled ‘‘Other.’’


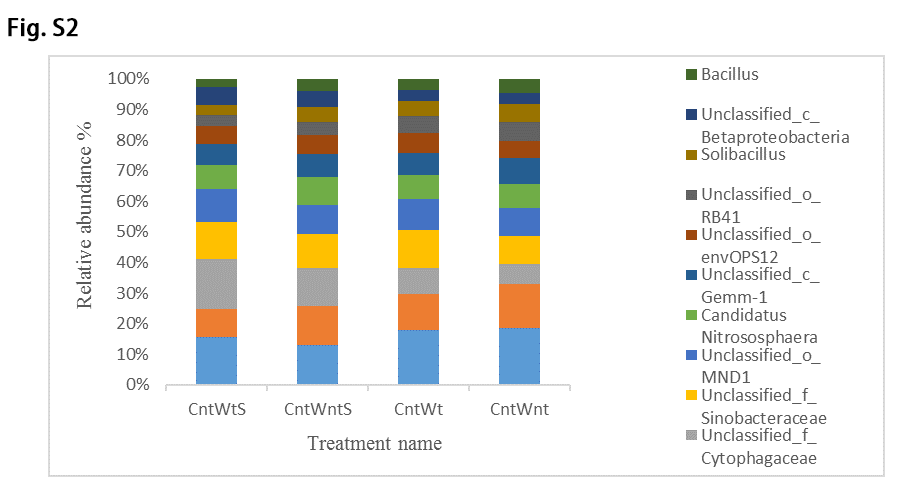


**Figure S2** Relative abundance of bacterial genera in four treatments. The values on the Y-axis show relative abundance (%) of classified and unclassified genera, and the X-axis shows different soil treatments.


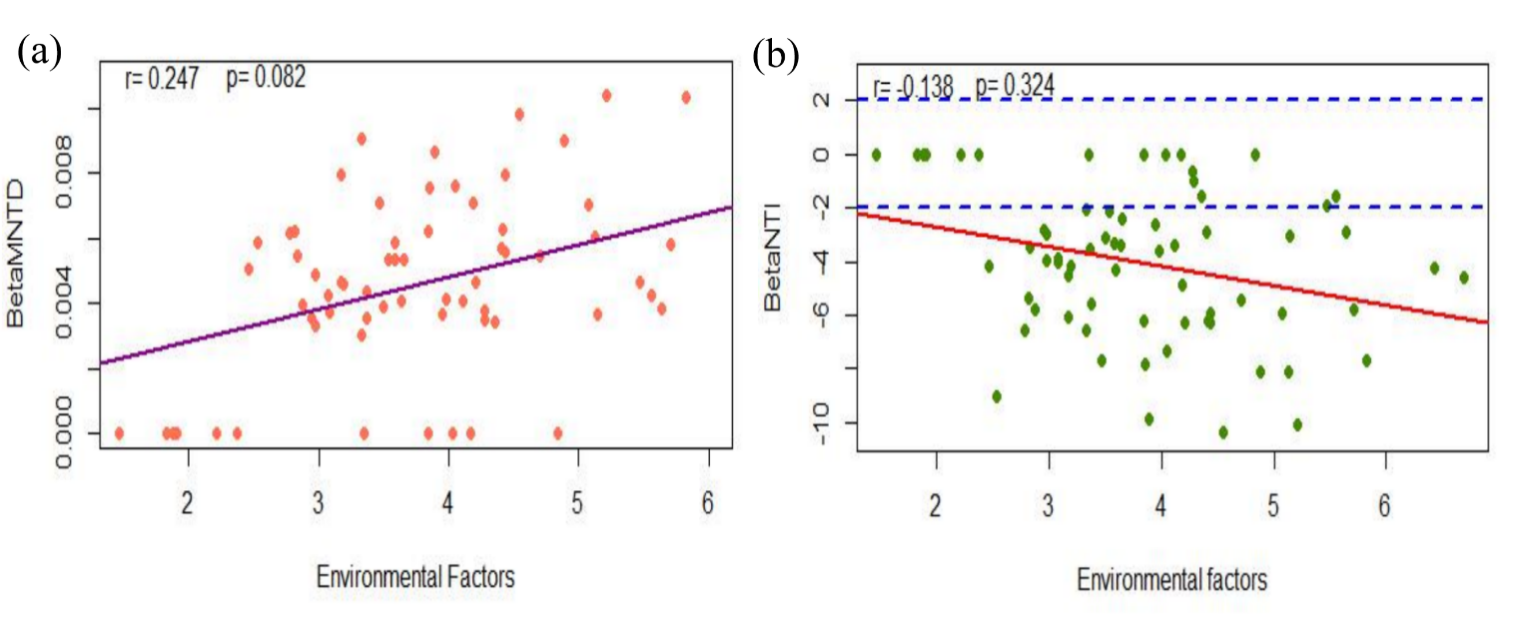


**Figure S3** Relationship among environmental distance, beta mean nearest taxon distance(betaMNTD) and beta nearest taxon index (betaNTI). The associated correlation coefficients and linear regression slop (shown as purple lines) are provided for each treatment. Horizontal blue dashed lines indicate lower (-2) and upper (+2) betaNTI significant thresholds.
